# SYNJ2BP promotes the degradation of PTEN through the lysosome-pathway and enhances breast tumor metastasis via PI3K/AKT/SNAI1 signaling

**DOI:** 10.18632/oncotarget.21058

**Published:** 2017-09-19

**Authors:** Miao Wang, Huijian Wu, Shujing Li, Zhaowei Xu, Xiahui Li, Yangyang Yang, Bowen Li, Yanan Li, Jing Guo, Huan Chen

**Affiliations:** ^1^ School of Life Science and Biotechnology, Dalian University of Technology, Dalian, China; ^2^ School of Life Science and Medicine, Dalian University of Technology, Panjin, China

**Keywords:** SYNJ2BP, PTEN, breast cancer, metastasis, lysosome

## Abstract

SYNJ2BP plays an important role in breast cancer metastasis. However, the molecular mechanism associated with the function of SYNJ2BP in metastasis remains unclear. In this study, we investigated the role of SYNJ2BP in tumor metastasis and established the associated underlying mechanism. Over-expression of SYNJ2BP promoted both cell migration and invasion. In contrast, silencing SYNJ2BP caused the suppression of cell migration and invasion. SYNJ2BP increased the levels of phosphorylation for AKT and GSK3β, which could be inhibited by the PI3K inhibitor, LY294002, and the GSK3β inhibitor, LiCl, and regulated the accumulation of SNAI1 in the nucleus and the expression of the SNAI1 target gene, E-cadherin (EMT marker). It is known that the stability of PTEN is regulated by ubiquitination. However, in this study, we additionally demonstrated that SYNJ2BP mediated the degradation of PTEN protein by the lysosome-pathway and induced the activation of PI3K/AKT signaling by promoting the co-localization of PTEN with autophagy-lysosomes and the expression of LC3-II and p62. In vivo study, the overexpression of SYNJ2BP significantly increased the metastasis of 4T1 cells in BALB/c mice. In addition, SYNJ2BP was highly expressed in breast carcinoma (*p* = 0.0031), but not in normal breast tissue, while analysis of tissue samples taken from SNAI1-positive human breast cancers showed a significant correlation between the expression of SYNJ2BP and that of p-AKT (*p* < 0.005). Collectively, our data identified a tumor inducer, SYNJ2BP, which could activate the PI3K/AKT/GSK3β/SNAI1 signaling pathway through the lysosome-mediated degradation of PTEN, and promote both EMT and tumor metastasis during the progression of breast cancer.

## INTRODUCTION

Cancer is a leading cause of death worldwide, and breast cancer is the most common cancer in females [1]. The metastasis of breast carcinoma is an important cause of treatment failure and patient mortality. Recent studies have shown that epithelial-mesenchymal transition (EMT) is a phenotypic transformation of epithelial cells into mesenchymal cells and plays a major role in metastasis [2]. In EMT, epithelial cells gain mesenchymal properties and exhibit reduced intercellular adhesion and increased motility [3]. One of the hallmarks of EMT is the loss of expression of both E-cadherin (a key epithelial marker of cell-cell adhesion) and the EMT transcription factor, SNAI1, which is the most important EMT inducer as it can bind to the E-Box and repress the expression of E-cadherin [4].

Phosphatase and tensin homologue deleted on chromosome 10 (PTEN) is a key player in the regulation of phosphatidylinositol 3-kinase (PI3K) signaling. The loss or mutation of PTEN in various human tumors can lead to hyperactive PI3K signaling [5]. Furthermore, the loss or abnormal degradation of PTEN is associated with multiple types of tumors, particularly breast tumors [6]. There are two major mechanisms responsible for intracellular degradation in mammalian cells, the ubiquitin proteasome pathway and the lysosome pathway. The ubiquitin-proteasome system is responsible for the highly selective degradation of misfolded or damaged proteins, and only unfolded ubiquitinated proteins can pass through the narrow pore of the proteasome barrel [7]. However, the large membrane proteins and protein complexes (including oligomers and aggregates) that fail to pass through the narrow proteasome barrel can be degraded by the autophagy-lysosome pathway. Two evolutionarily-conserved protein conjugation systems are necessary for the formation of the autophagosome, the autophagy related (ATG)12-ATG5- and the ATG8-phosphatidylethanolamine conjugation systems. The ATG8 homologue is known as light chain 3 (LC3), and is cleaved by ATG4 to expose a C-terminal residue, which defines LC3-II and is strongly associated with the autophagosomal membrane via p62 [8]. It has been established that the autophagy-lysosome pathway is a non-selective degradation pathway. However, it is not clear whether specific protein substrates are targeted directly for degradation by the autophagy-lysosome pathway.

PI3K converts phosphatidylinositol bisphosphate [PI(4,5)P2] to phosphatidylinositol triphosphate [PI(3,4,5)P3], a second messenger that activates intracellular AKT signaling pathways [9]. PI(3,4,5)P3 can be dephosphorylated by Synaptojanin 2 (SH2-containing inositol polyphosphate 5-phosphatase, SYNJ2) to yield PI(3,4)P2, and SYNJ2 recruits TKS5 and forms new invadopodia resulting in increasing tumor invasiveness [10]. The PI3K/AKT signaling pathway is a major regulator in the activation of glycogen synthase kinase-3 beta (GSK-3β) [11]. The phosphorylation of GSK3β by AKT promotes the ubiquitination and degradation of GSK3β. The dephosphorylation and activation of GSK3β also increases SNAI1 phosphorylation and nuclear export, which are essential processes for ubiquitin-mediated degradation [12]. Phosphorylation of GSK3β stabilizes SNAI1 and enhances the SNAI1-mediated repression of E-cadherin expression. However, only the nuclear localization of SNAI1 increases its activation and promotes EMT [13]. Hence, the PI3K/AKT/GSK3β pathway can affect EMT and subsequently influence the aggressiveness of tumors.

Synaptojanin-2 binding protein (SYNJ2BP), also known as ARIP2 and OMP25, acts as a binding protein for SYNJ2 and Activin type II receptors (ActR-II), and the cellular localization of SYNJ2 and ActR-II is regulated by SYNJ2BP [14, 15], which has a single PDZ domain at the N-terminus. PDZ domains are protein-interaction domains that are often found in multi-domain scaffolding proteins [16]. PDZ-containing proteins modify themselves in order to influence the function, stabilization, or location of the transmembrane [17]. There is a transmembrane region near the C-terminus of SYNJ2BP, which helps to promote localization to the cytoplasmic membrane or mitochondria outer membrane [14, 15]. SYNJ2BP expression is much more frequent in malignant breast carcinoma [17], and may play an important role in the progression of cancer [18], However, little is known about the precise mechanisms underlying the effect of SYNJ2BP expression in tumor metastasis.

In this study, we found that SYNJ2BP was activated via the PI3K/AKT/GSK3β/SNAI1 signaling pathway by the lysosome-mediated degradation of PTEN (a novel degradation mechanism of PTEN), which then resulted in changes in the EMT phenotype and the subsequent promotion of invasiveness and metastasis of breast cancer cells *in vivo* and *vitro*. Moreover, SYNJ2BP expression levels in breast carcinoma patients was statistically associated with tumor stage. Our data thus identified a potential tumorigenesis role for SYNJ2BP in breast cancer.

## RESULTS

### SYNJ2BP levels correlate with the progression of breast cancer

**Figure 1:**
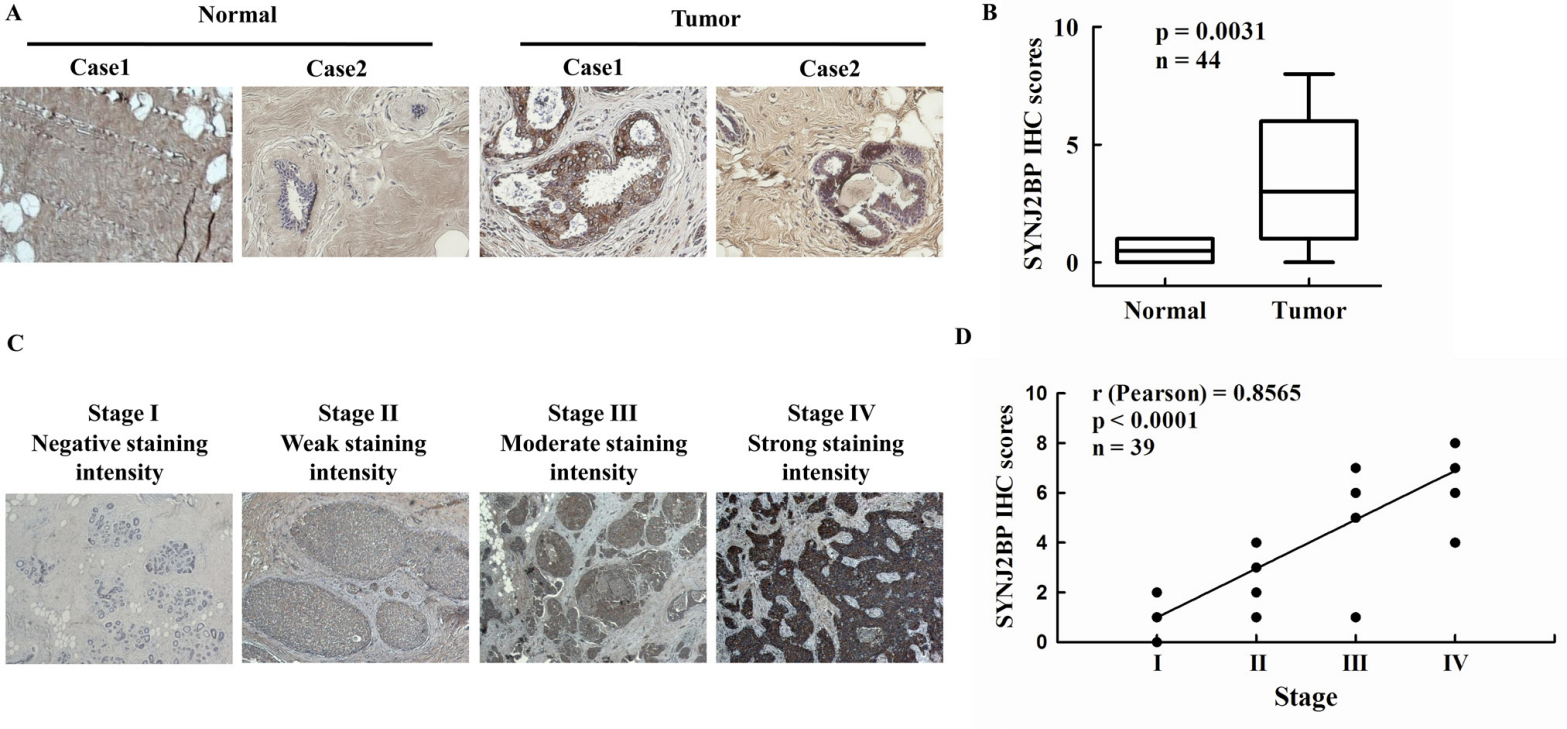
The expression of SYNJ2BP in breast tumor tissue samples (**A**) Immunohistochemistry analysis of SYNJ2BP expression in normal breast tissues and breast tumor tissues. (**B**) Expression levels of SYNJ2BP in breast cancer sample (*n* = 39) and normal breast sample (*n* = 5). Box indicates the median and the interquartile range, and whiskers represent minimum and maximum. (**C**) Immunohistochemistry analysis of SYNJ2BP expression in breast cancer progression from TNM stage I to IV. (**D**) The correlation of SYNJ2BP level and tumor stage in breast tumors. The r and *p*-values were obtained by Pearson's correlation analysis.

In order to determine the clinical significance of SYNJ2BP in breast cancer patients, we analyzed the expression levels of SYNJ2BP in 5 normal (non-tumor) human breast tissues and 39 breast carcinoma tissues by immunohistochemical (IHC) staining. A IHC score of 0 to 8 (0 = negative or no expression; 1–2 = weak expression, < 10%; 3–5 = moderate expression, between 10 and 50%; and 6 - 8 = high expression, > 50%) was assigned to the expression level of SYNJ2BP in the cells. Every section was scored according to the proportion (%) of stained tumor cells and the staining intensity. According to IHC score, SYNJ2BP was expressed at higher levels in breast cancer tissues than in normal breast tissues, and a *t*-test identified a significant difference between the two groups ( *p* = 0.0031; Figure 1A and 1B). Accordingly, the level of SYNJ2BP expression was positively correlated with the development of breast tumors.

To validate the correlation between SYNJ2BP expression and tumor progression, immunohistochemical (IHC) analysis of SYNJ2BP was performed in a cohort of 39 human breast tumors. SYNJ2BP was detected in 28 out of the 39 (71%) tumors. Interestingly, 6 out of the 39 (15%) tumors showed very high (> 6) SYNJ2BP immunoreactive scores, which were closely related to tumor progression from TNM stage IV. Intra-tumoral SYNJ2BP levels increased gradually with tumor progression from TNM stage I to IV (Figure 1C). Furthermore, Pearson's correlation analysis identified a positive correlation between SYNJ2BP and the tumor stage of breast cancer (r = 0.8565; Figure 1D). These data indicated that high intra-tumoral SYNJ2BP expression may be an indicator of breast cancer progression.

### SYNJ2BP expression improves cellular migration and invasion in breast cancer

**Figure 2:**
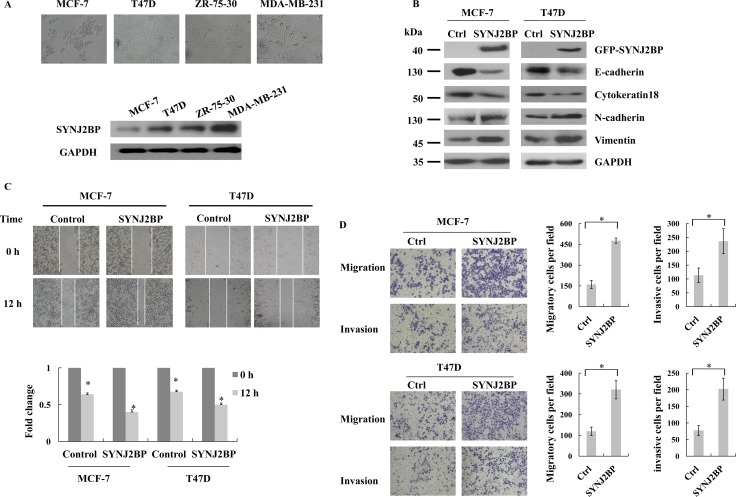
The effects of SYNJ2BP expression on breast cancer cellular EMT phenotype (**A**) Phase-contrast microscopic images of the human breast cancer cell lines MCF-7, T47D, ZR-75-30, MDA-MB-231 (up), and western blot analysis of SYNJ2BP expression in these cells (down). (**B**) Western blot showing the effects of SYNJ2BP overexpression on the expression levels of E-cadherin, Cytokeratin 18, N-cadherin and Vimientin in MCF-7 and T47D cells. (**C**) Scratch wound-healing assay assessing the effects of SYNJ2BP overexpression on the motility of MCF-7 and T47D cells. The bar graph shows the fold change of 12 h compare with 0 h wound-healing, 0 h is set to 1. (**D**) Trans-well migration and invasion assay evaluation the effects of SYNJ2BP overexpression on the cellular motility and invasion ability of MCF-7 and T47D cells. The bar graph shows the number of migrating and invading cells for each category of cells (right).

To determine the relevance of SYNJ2BP function in the progression of breast cancer, we investigated the expression of SYNJ2BP in four different human breast cancer cell lines. The order of increasing metastatic potential for the four cell lines was MCF-7<T47D<ZR-75-30<MDA-MB-231. Low levels of SYNJ2BP expression were found in MCF-7 and T47D cells, which displayed epithelioid morphology and low metastases. However, high levels of SYNJ2BP expression was found in ZR-75–30 and MDA-MB-231cells, both of which are high metastatic cells with mesenchymal-like morphology (Figure 2A). This suggested that SYNJ2BP plays an important role in the regulation of cell morphology and the progression of breast cancer.

Recently, EMT has been shown to be positively correlated with the metastatic potential of tumor cells, suggesting that it plays a fundamental role in tumor invasion and metastasis [19]. In the present study, we examined the expression levels of epithelial and mesenchymal markers in SYNJ2BP-overexpressing cells to evaluate the effect of SYNJ2BP on cellular EMT. The overexpressing SYNJ2BP in MCF-7 and T47D cells reduced the levels of the epithelial markers, E-cadherin and Cytokeratin 18, but increased the levels of the mesenchymal markers, N-cadherin and Vimentin (Figure 2B). These data suggested that SYNJ2BP may have an important role in the expression of EMT markers.

Next, we used scratch wound-healing and trans-well assays to demonstrate the migration and invasion capacity of breast cancer cells. Overexpressing SYNJ2BP in MCF-7 and T47D cells led to enhanced cell motility, and a larger proportion of cells were able to migrate through the Matrigel-coated membrane, compared to control cells (Figure 2C and 2D). These results suggested that SYNJ2BP may play a key role in the promotion of mobility and invasiveness, which may possibly be connected to its effect on the organization of the cytoskeleton in breast cancer cells.

### SYNJ2BP ablation suppresses the cellular migration and invasion of breast cancer

**Figure 3:**
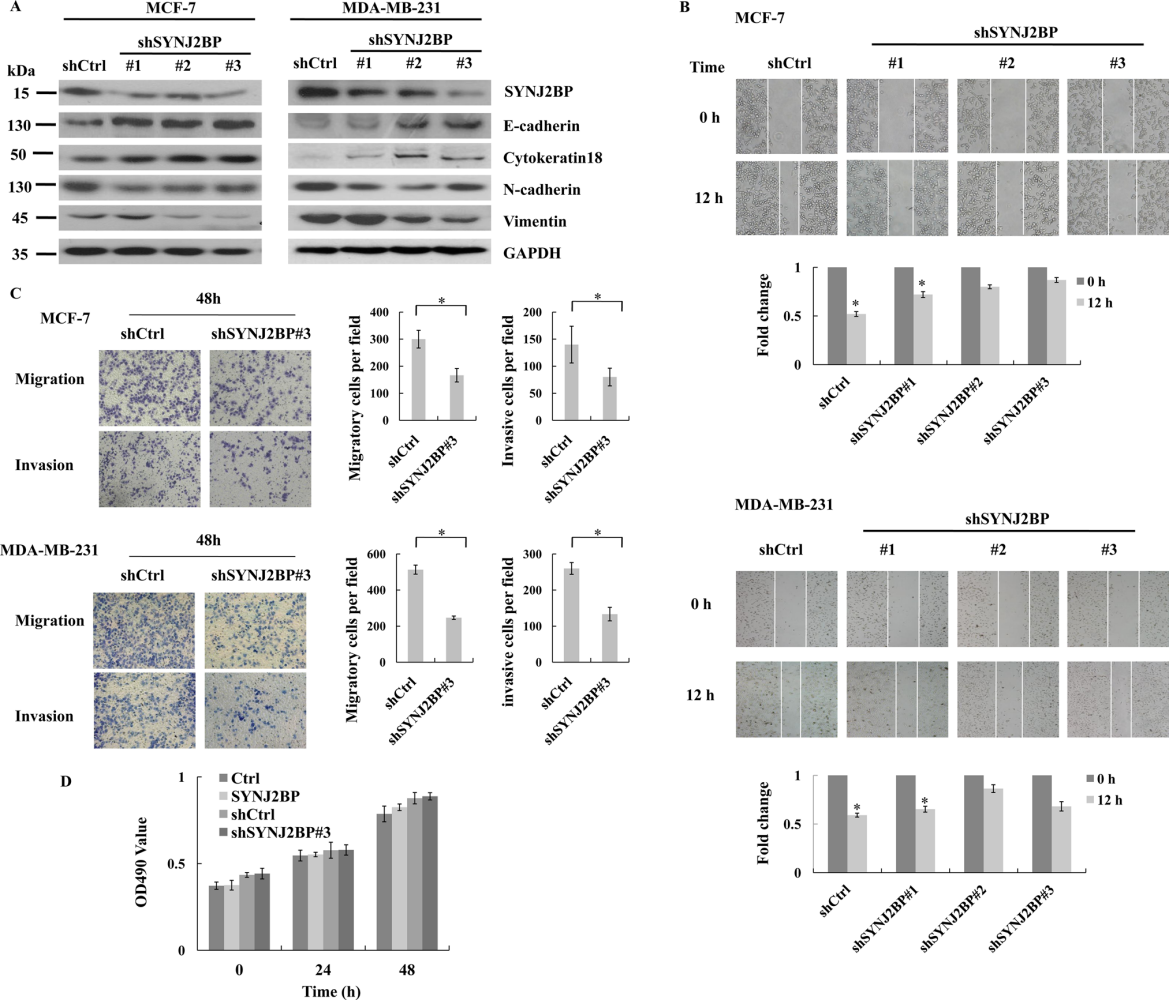
The effects of SYNJ2BP ablation on breast cancer cellular EMT phenotype (**A**) Western blot showing the effects of SYNJ2BP knockdown on the expression levels of E-cadherin, N-cadherin, Vimientin in MCF-7 and MDA-MB-231 cells. (**B**) Scratch wound-healing assay assessing the effects of SYNJ2BP knockdown on the motility of MCF-7 and MDA-MB-231 cells. The bar graph shows the fold change of 12 h compare with 0 h wound-healing, 0 h is set to 1. (**C**) Trans-well migration and invasion assay evaluation the effects of SYNJ2BP knockdown on the cellular motility and invasion ability of MCF-7 and MDA-MB-231 cells. The bar graph shows the number of migrating and invading cells for each category of cells (right). (**D**) MTT assays showing the effect of SYNJ2BP on cell proliferation in MCF-7 cells within 24 and 48 hours.

To further confirm the crucial role of SYNJ2BP in EMT in breast cancer cells we expressed shRNA (shSYNJ2BP#1, #2, and #3) to reduce the expression of SYNJ2BP in MCF-7 and MDA-MB-231 cells and examined the expression level of epithelial and mesenchymal markers. SYNJ2BP knockdown increased the expression levels of E-cadherin and Cytokeratin 18 in MCF-7 and MDA-MB-231 cells. However, SYNJ2BP knockdown reduced the expression levels of N-cadherin and Vimentin in MCF-7 and MDA-MB-231 cells (Figure 3A). The migration and invasion capacity of these cells were further demonstrated by wound-healing and trans-well assays. MCF-7 and MDA-MB-231 cells which expressed shSYNJ2BP#3 showed a lack of cell motility and migration capacity, compared to control cells (Figure 3B and 3C). These data suggested that the disruption of SYNJ2BP expression inhibited breast cancer cell migration and invasion, by regulating the expression of EMT markers.

In order to confirm that the higher numbers of migrating and invading cells observed for cells that overexpressed SYNJ2BP were not a result of an increase in cell proliferation caused by SYNJ2BP, we carried out an MTT assay to compare differences in cell proliferation among control cells, MCF-7 cells that overexpressed SYNJ2BP, and MCF-7 cells in which SYNJ2BP had been knocked-down. No significant differences were observed in cell proliferation among these different groups of cells and no significant increases were detected at 24 h and 48 h (Figure 3D). This showed that the effect of SYNJ2BP on cell proliferation within a 48-h period was insignificant, and that the overexpression of SYNJ2BP, and the stronger migration displayed by these cells compared to SYNJ2BP knockdown cells, were not due to cell proliferation caused by SYNJ2BP. Collectively, these results demonstrated that SYNJ2BP may have an important regulatory role in cell motility and invasion, and that this role is not influenced by its effect on cell proliferation.

### SYNJ2BP regulates EMT through PI3K/AKT/GSK3β/SNAI1 signaling

**Figure 4:**
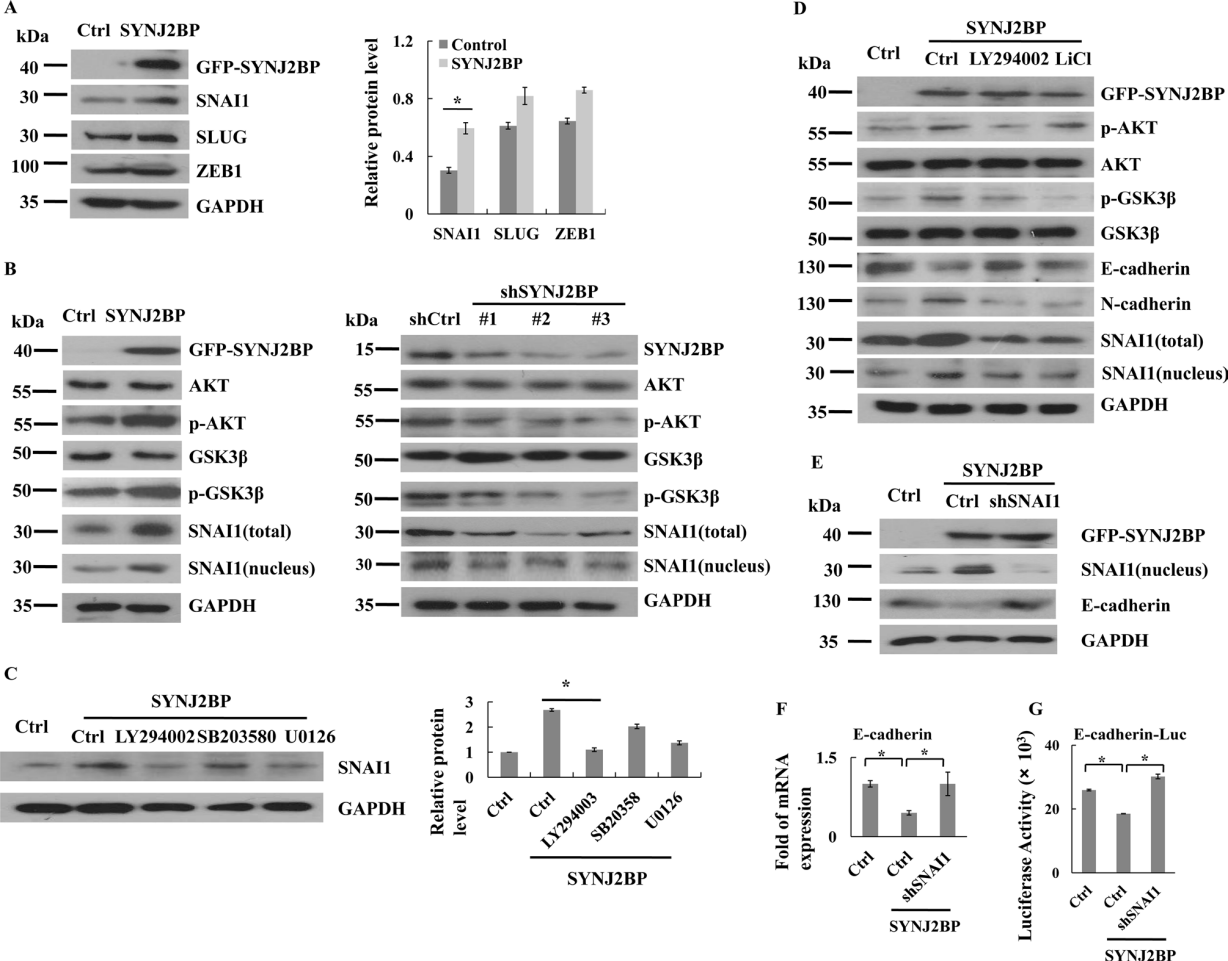
SYNJ2BP regulates breast cancer cellular EMT via PI3K/AKT/GSK3β/SNAI1 (**A**) Western blots showing that effects of SYNJ2BP overexpression on the expression levels of SNAI1, SLUG, ZEB1. The bar graph showing the relative band intensity of SNAI1, SLUG, ZEB1 expression levels normalized to GAPDH (right). (**B**) Western blots showing that effects of SYNJ2BP overexpression (left) or knockdown (right) on the expression levels of AKT, pAKT, GSK3β, pGSK3β, SNAI1 and pSNAI1. (**C**) Western blot showing that effects of SYNJ2BP overexpression with or without LY294002 (24 nM) or U0126 (10 mM) or SB203580 (20 mM) treatment on the expression levels of SNAI1. (**D**) Western blot showing that effects of SYNJ2BP overexpression with or without LY294002 (24 nM) or LiCl (10 mM) treatment on the expression levels of AKT, pAKT, GSK3β, pGSK3β, SNAI1, E-cadherin and N-cadherin in MCF-7 cells. (**E**) Western blot analysis showing the reversion of repressed E-cadherin expression in SNAI1-knockdown cells by overexpression of SYNJ2BP. MCF-7 cells without or with SNAI1 knockdown were transfected with SYNJ2BP and the levels of SNAI1, E-cadherin and GAPDH in the cells were analyzed by western blot. RT-PCR analysis (**F**) and reporter-gene assay (**G**) showing the level of E-cadherin transcript in the different groups of cells in E.

As SYNJ2BP could reduce the expression of E-cadherin, it was important to investigate the underlying mechanism. We analyzed the expression levels of several known E-cadherin transcriptional repressors in MCF-7 cells that overexpressed SYNJ2BP. The overexpression of SYNJ2BP resulted in significant upregulation of SNAI1 expression, but caused little change in the expression of SLUG and ZEB1 (Figure 4A). Nuclear accumulation of SNAI1 caused an increase in the inhibition of E-cadherin expression. As shown by SYNJ2BP-overexpressing cells, the nuclear accumulation and transcriptional activity of SNAI1 were increased, and the expression of E-cadherin was inhibited (Figures 4B and 2B).

The phosphorylation of GSK3β induced its inactivation and ubiquitin-proteasome degradation. Therefore, the phosphorylation of GSK3β reduced the ubiquitination and degradation of SNAI1. To test whether the activation of SNAI1 resulting from the overexpression of SYNJ2BP was mediated by the modulation of GSK3β activity, we analyzed both the expression and phosphorylation of GSK3β and the subcellular localization of SNAI1. We found that GSK3β phosphorylation at Ser9 was significantly increased in SYNJ2BP-overexpressing cells, and at the same time, this also led to increased SNAI1 stability (Figure 4B, left). In contrast, knockdown of SYNJ2BP expression through shRNA (shSYNJ2BP#1, #2, and #3) reduced GSK3β phosphorylation and increased the transportation of SNAI1 from the nucleus to the cytoplasm for degradation (Figure 4B, right). These results indicated that SYNJ2BP-mediated SNAI1 activation was dependent on the phosphorylation of GSK3β.

As GSK3β activity can be regulated by the PI3K, ERK1/2, and p38 pathways [20], it was first necessary to determine which particular signaling pathway is involved in SYNJ2BP-induced SNAI1 activation. SYNJ2BP-overexpressing cells, treated with a specific inhibitor to block each of these signaling pathways, showed that increased accumulation of SNAI1 in the nucleus was related to SYNJ2BP overexpression, and that SNAI1 expression was only notably inhibited by the PI3K inhibitor LY294002 (Figure 4C); there was no significant effect in response to the ERK1/2 inhibitor, U0126, and the p38 inhibitor, SB203580. These findings suggested that the PI3K pathway is critical in the SYNJ2BP-induced increase in the level of SNAI1 protein in the nucleus. In support of this notion, we also found that the activity of AKT, a downstream effector of PI3K, was upregulated in SYNJ2BP-overexpressing cells compared to control cells (Figure 4B, left). In contrast, the knockdown of SYNJ2BP expression through shRNA (shSYNJ2BP#1, #2, and #3) reduced AKT phosphorylation (Figure 4B, right). Moreover, the PI3K inhibitor, LY294002, and the GSK3β inhibitor, LiCl, could alleviate the EMT phenotype of MCF-7 cells induced by SYNJ2BP overexpression, leading to reduced expression of E-cadherin, and increased levels of N-cadherin and accumulation of SNAI1 in the nucleus (Figure 4D). Collectively, these results suggested that SYNJ2BP promoted SNAI1 activation, and downregulated E-cadherin expression, predominantly through the activation of the PI3K/AKT pathway.

For MCF-7 cells overexpressing SYNJ2BP, the level of SNAI1 expression in the nucleus was markedly reduced, at least by more than 80% in cells with SNAI1 knockdown compared to control cells (Figure 4E). The higher level of SNAI1 expression in the nucleus caused by the overexpression of SYNJ2BP was also supported by the lower SNAI1 expression in MCF-7 cells that did not overexpress SYNJ2BP, but without SNAI1 knockdown. As for E-cadherin, transcript and protein levels were both increased in SNAI1-knock down MCF-7 cells that were overexpressing SYNJ2BP, compared to cells that did not overexpress SYNJ2BP, and were without SNAI1 knockdown, and this reaffirmed that the difference in E-cadherin expression was caused by SYNJ2BP (Figure 4E–4G). These data again demonstrated that SYNJ2BP-induced EMT was dependent on SNAI1 activation through the PI3K/AKT/GSK3β pathway.

### SYNJ2BP promotes the degradation of PTEN

**Figure 5:**
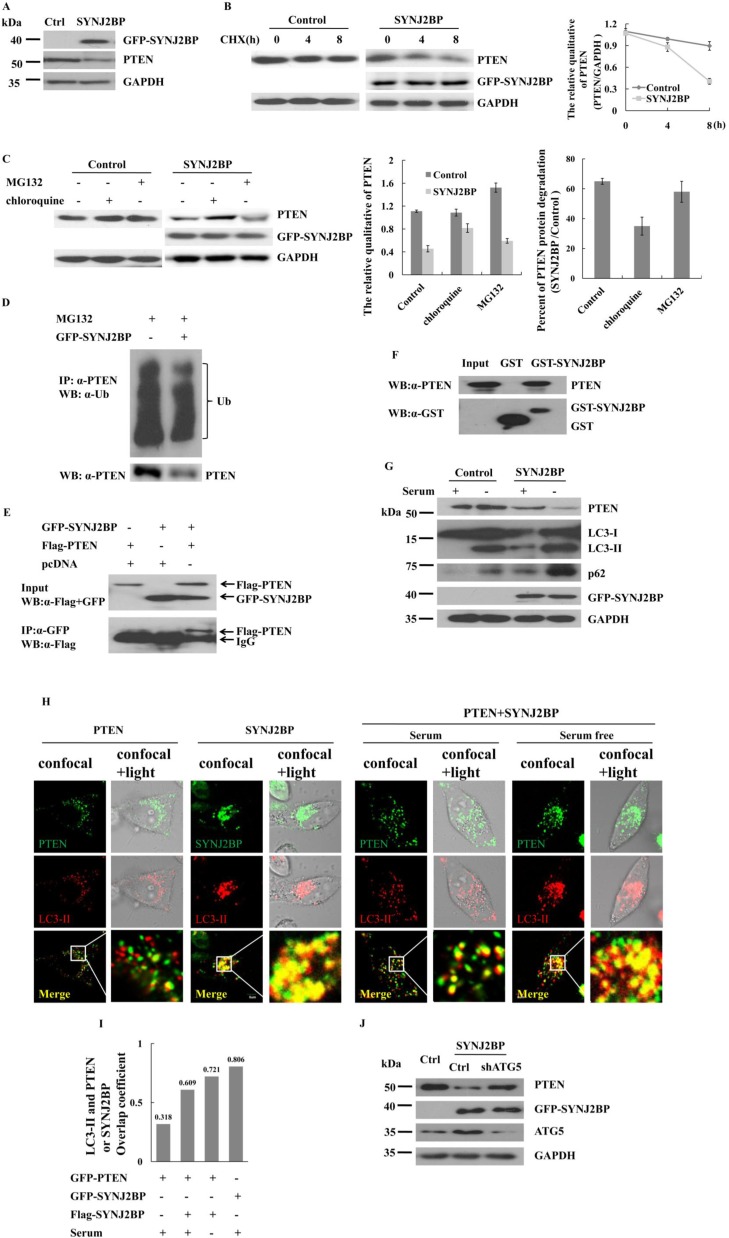
Effects of SYNJ2BP on the expression of PTEN (**A**) Western blot showing that effects of SYNJ2BP overexpression on PTEN expression. Cells were transfected with GFP- SYNJ2BP or control vector and collected after 22 h. (**B**) Western blot showing that effects of SYNJ2BP overexpression on PTEN level. Cells transfected with GFP- SYNJ2BP or control vector and treated with 10 mg/ml cycloheximide (CHX) for different periods of time (0, 4, 8 h) before being subjected to western blot. The graph shows the relative intensity of the PTEN band at different time points. The level of PTEN protein for 0 h in control or SYNJ2BP cells was set to 1, respectively. (**C**) Western blot showing that effects of SYNJ2BP overexpression on PTEN degradation. MCF-7 cells transfected with GFP-SYNJ2BP or control vector and treated without or with 10 mM MG132 or 50 μM chloroquine for 8 h. The samples were subjected to western blot analysis with the indicated antibodies. Quantified protein levels of PTEN (relative to GAPDH) was plotted in the left bar graph. SYNJ2BP-induced PTEN degradation by lysosome and proteasome pathway were plotted by a percentage in the right bar graph. (**D**) Co-IP assay showing that effects of SYNJ2BP overexpression in PTEN ubiquitylation. **(E**) Co-IP assay showing the interaction between exogenous SYNJ2BP and PTEN. MCF-7 cells were cotransfected with Flag-PTEN and GFP-SYNJ2BP. After 24 h of transfection, the cells were subjected to immunoprecipitation with anti-GFP antibody, followed by western blot analysis with anti-GFP antibody. (**F**) GST-pull down assay showing the interaction between GST-SYNJ2BP and endogenous PTEN. (**G**) Western blot showing that effects of SYNJ2BP overexpression on PTEN, LC3 and p62 level. Cells were transfected with GFP-SYNJ2BP or control vector for 22 h and then treated with or without serum 2 h before lyses. (**H**) Subcellular localization of PTEN or SYNJ2BP shown by confocal fluorescence microscopy. MCF-7 cells were transfeced with GFP-PTEN or GFP-SYNJ2BP alone or Flag-PTEN and GFP-SYNJ2BP and treated with or without serum. LC3-II was used to indicate autophagy-lysosome. Merged images are shown in bottom panels. The merge of confocal and transmitted light showing protein distribution of inside the cells (right). (**I**) Quantification of the colacalization between PTEN and LC3-II in control or SYNJ2BP overexpression cells and treated with or without serum, using overlap coefficient. (**J**) Western blot analysis showing the reversion of repressed PTEN expression in ATG5-knockdown cells by overexpression of SYNJ2BP. MCF-7 cells without or with ATG5-knockdown were transfected with SYNJ2BP or control and the levels of PTEN, ATG5 and GAPDH in the cells were analyzed by western blot.

As the activation of PI3K/AKT signaling may be associated with the loss of *PTEN* [9, 21, 22], we examined the protein level of endogenous PTEN to evaluate the effect of SYNJ2BP in PTEN-positive MCF-7 cells. The overexpression of SYNJ2BP was found to reduce the expression levels of PTEN (Figure 5A).

The level of a protein within a cell is determined by the rate of its synthesis or degradation. The expression of PTEN in SYNJ2BP-overexpressing cells was examined following treatment with cycloheximide (CHX), a synthesis inhibitor. The level of PTEN in SYNJ2BP-overexpressing cells was reduced by 45% compared to control cells just 8 h after treatment with CHX (Figure 5B). A reduction in the expression of a protein usually involves an increase in its degradation via the ubiquitin-proteasome or autophagy-lysosome pathway. Lysosomes are responsible for the degradation of membrane or extracellular proteins that enter the cells by endocytosis [23], and SYNJ2BP is known to be associated with endocytosis. To determine what extent the proteasome, or lysosomes, contributed to the SYNJ2BP-induced degradation of PTEN, we treated cells with either a specific proteasomal inhibitor (MG132), or a specific lysosomal inhibitor (chloroquine), and then determined the expression of PTEN. Our results showed that in control cells, PTEN degradation was inhibited by MG132, but not by chloroquine. In contrast, PTEN degradation was mainly inhibited by chloroquine in SYNJ2BP-overexpressing MCF-7 cells (Figure 5C, left and middle panels). The rates of PTEN degradation in control cells increased by 65% compared to that of cells overexpressing SYNJ2BP. This increase was reduced to less than 10% after treatment with a proteasome inhibitor, but was reduced to 50% after treatment with a lysosomal inhibitor (Figure 5C, right panel). Surprisingly, this level of reduction was only marginally inhibited by proteasome inhibitors, but was markedly reduced by lysosomal inhibitors. In fact, approximately 50% of the increase in degradation required lysosomal activity, while only 10% required proteasome activity in SYNJ2BP-overexpressing cells.

Most proteins require the formation of polyubiquitin chains in order to be degraded by the 26S proteasome. To investigate whether SYNJ2BP would promote PTEN degradation and polyubiquitination, we used MG132 to block the degradation of protein followed by subsequent Co-IP assay. Data revealed no change in the polyubiquitin chain of PTEN in SYNJ2BP overexpressing cells, suggesting that SYNJ2BP did not promote PTEN ubiquitination, even though PTEN protein levels fell sharply, by almost 50% (Figure 5D). Thus, stabilization of the PTEN protein appeared to be regulated by the ubiquitin-proteasome pathway, but there may also be another pathway through which PTEN can be degraded in SYNJ2BP-overexpressing cells. Furthermore, this alternative PTEN degradation pathway was blocked by a lysosomal inhibitor, but not by a proteasomal inhibitor, suggesting that SYNJ2BP played a role in both endocytosis and lysosomal degradation. To explore the mechanism of SYNJ2BP-regulated lysosome-mediated PTEN degradation, we performed co-immunoprecipitation and GST pull-down assays to investigate the interaction between SYNJ2BP and PTEN; data showed that PTEN co-precipitated with SYNJ2BP, suggesting that the two proteins probably interacted with each other *in vitro* (Figure 5E) and *in vivo* (Figure 5F).

Activation of the lysosomal degradation pathway was further confirmed by Western blot analysis of p62 and microtubule-associated protein light-chain 3 (LC3). LC3-II is conjugated to phosphatidylethanolamine during lysosome turnover and during autophagic lysosomal vacuole formation. P62 is an autophagic adaptor, which contains an LC3-interacting region that recognizes molecules and assists them into autophagy [24]. Thus, the presence of LC3-II and p62 reflects the existence of starvation-induced autophagic-lysosome activity [25]. The overexpression of SYNJ2BP was associated with the expression of p62, LC3-I and LC3-II (Figure 5G) and accompanied by a 60% increase in PTEN degradation (Figure 5C). SYNJ2BP-overexpressing cells exhibited significant increases in lysosomal degradation than non-SYNJ2BP-overexpressing cells, especially following serum starvation (Figure 5G). These results suggested that increased levels of SYNJ2BP expression in the cells could induce the degradation of PTEN via the lysosomal pathway.

Next, the subcellular location of PTEN and LC3-II were determined via confocal microscopy. The co-localization of PTEN with LC3-II increased by almost double in SYNJ2BP-overexpressing cells (Figure 5H, left panel). However, the co-localization of PTEN with LC3-II in SYNJ2BP-overexpressing cells was enhanced by 1.5-fold following serum starvation (Figure 5H, right panel and 5I). In SYNJ2BP overexpressing cells, there was still a fraction of PTEN localized in the periphery of cells under normal serum culture conditions. The co-localization of PTEN with LC3-II was enhanced by serum starvation in SYNJ2BP-overexpressing cells, with most of the PTEN distributed around the nucleus. Moreover, confocal images also showed strong co-localization of SYNJ2BP with LC3-II, implying the existence of SYNJ2BP-mediated co-localization of PTEN with the autophagosome in MCF-7 cells.

ATG5 and LC3 (ATG8) protein conjugation systems are also necessary for the formation of the autophagosome [26]. Whether PTEN degradation is caused by autophagy, we analyzed PTEN expression by knocking-down the autophagy gene ATG5 in SYNJ2BP-overexpressing cells. In cells which were overexpressing SYNJ2BP, the level of PTEN expression increased with ATG5 knockdown compared to control cells (Figure 5J). These data again demonstrated that SYNJ2BP induced PTEN degradation in a manner which was dependent on the autophagic-lysosome pathway.

### SYNJ2BP increases breast cancer cell metastasis *in vivo*

**Figure 6:**
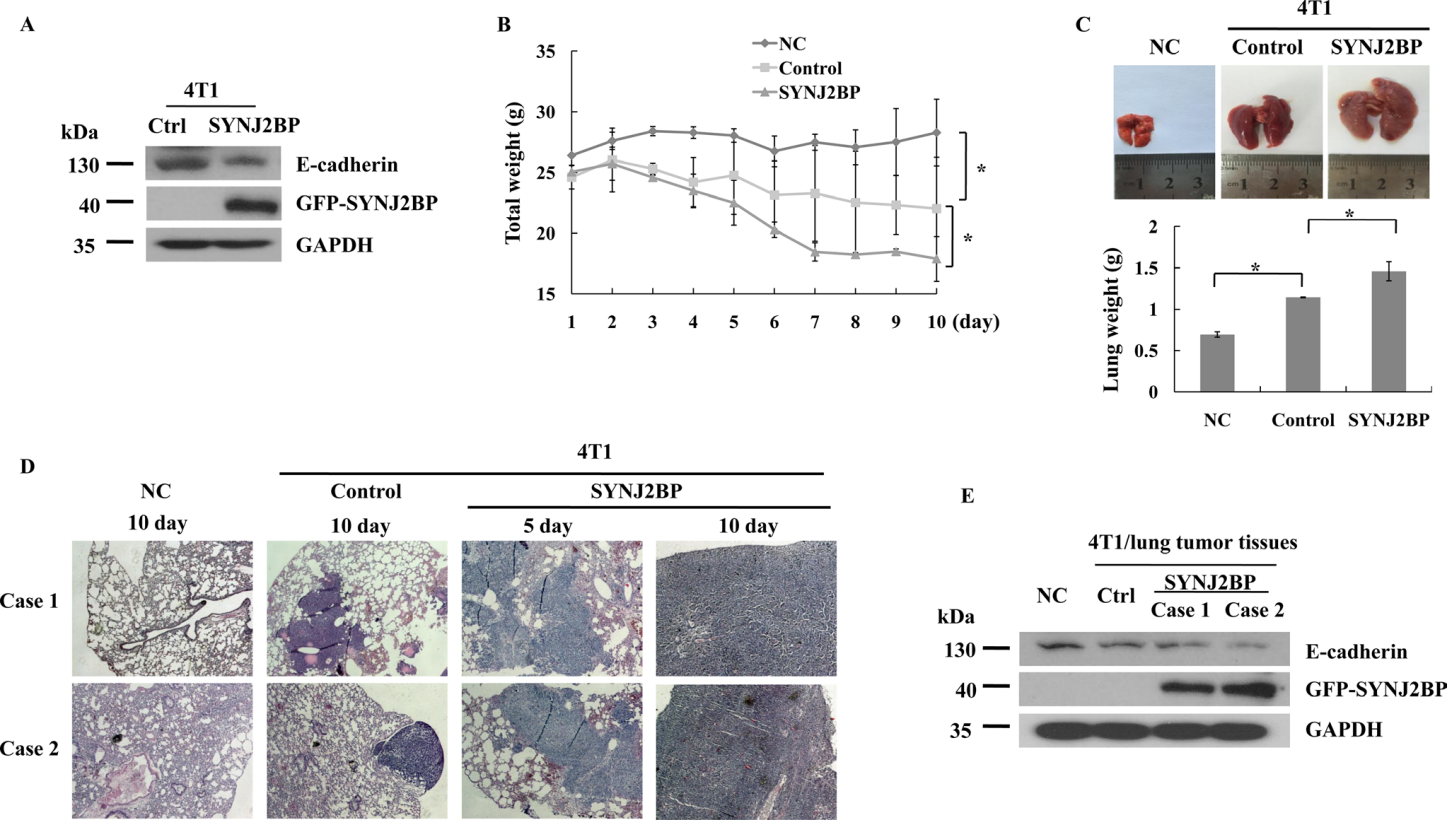
SYNJ2BP modulates the metastasis and growth of 4T1 cells in BALB/c mice (**A**) Western blot showing that effects of SYNJ2BP overexpression on the expression levels of E-cadherin in 4T1 cells. Mice injected with SYNJ2BP overexpressed cells. Mice weight (**B**), lungs images and lungs weight (**C**) 10 days after mice were injected subcutaneously with 4T1 cells overexpressed SNYJ2BP or empty vector. (**D**) Lungs from mice injected with normal saline (negative control, NC), 4T1 cells (control) and 4T1 cells overexpressed SYNJ2BP 5 or 10 days were isolated and stained with H&E to determine cancer metastasis. (**E**) Expression level of E-cadherin was examined by western blot in control and SYNJ2BP overexpression 4T1 lung tumor tissues. *n* = 5 mice per group in B, C, D and E.

The overexpression of SYNJ2BP reduced E-cadherin expression in 4T1 cells (Figure 6A). Thus, the correlation between SYNJ2BP and metastasis was further investigated in BALB/c mice. BALB/c mice injected with 4T1 cells that stably overexpressed SYNJ2BP showed significant increases in lung metastasis compared to mice injected with 4T1 that were stably transfected with the empty vector (control), or those injected with just normal saline (negative control, NC). Mice injected with 4T1 cells that overexpressed SYNJ2BP showed significant weight loss from day 5 onwards and such weight loss continued until the end of the experiment (Figure 6B). The size and weight of the lungs from BALB/c mice that overexpressed SYNJ2BP were significantly increased compared to the control and NC mice (Figure 6C). Furthermore, histological analysis, using hematoxylin and eosin (HE) staining, showed that the metastatic 4T1 cells had colonized the lung. In mice that were injected with 4T1 cells that overexpressed SYNJ2BP, multiple local tumor growths of densely clustered tumor cells were seen in the lung, which significantly increased tumor infiltration into the metastatic lesions from the 5th day. In contrast, tumor cells in mice that were injected with control 4T1 cells almost spread to the entire animal by the 10th day (Figure 6D). This implied that the metastasis of cancer cells from the lung was a consequence of SYNJ2BP overexpression. Taken together, these data showed that SYNJ2BP could enhance breast cancer cell metastasis in an animal model, and that SYNJ2BP could act as a crucial factor in the promotion of breast cancer cell metastasis.

**Figure 7:**
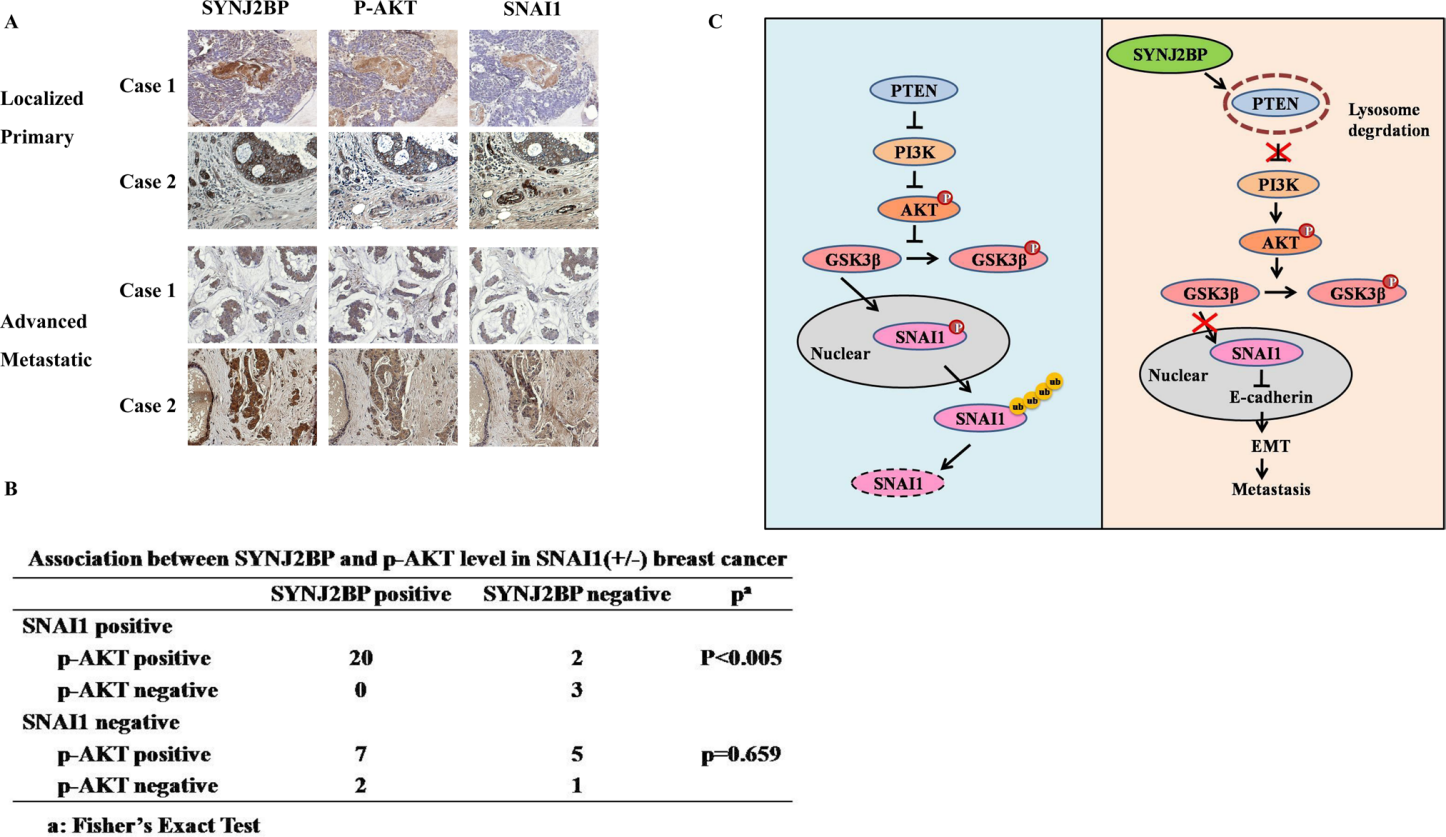
Correlation between SYNJ2BP, p-AKT and SNAI1 expressions in human breast tissue samples (**A**) Representative examples of immunohistochemical staining for SYNJ2BP, p-AKT, SNAI1 in normal human breast tissues and breast carcinoma tissues as indicated. (**B**) Association between SYNJ2BP and p-AKT levels in SNAI1 (+/ −) breast cancer tissue samples (*n* = 39). Significance was determined using a two-sided Fisher's exact test (*P* < 0.005). (**C**) Propose model of SYNJ2BP signaling pathway in EMT and metastasis.

To further determine the *in vivo* relevance of our data obtained by cell culture, the expression levels of SYNJ2BP, p-AKT and SNAI1 of five normal human breast tissues and 39 breast carcinoma tissues were analyzed by IHC. The expression of SYNJ2BP was low in normal breast tissues, but increased in breast tumor tissues (Figure 7A). The advanced metastatic tissues displayed high SYNJ2BP staining with concomitant higher cytoplasm p-AKT staining and higher nuclear SNAI1 staining than normal breast tissue. Moreover, a significant (*p* < 0.005) correlation between SYNJ2BP and p-AKT was also observed in SNAI1-positive breast cancer tissue samples (Figure 7B). These findings were consistent with our speculation that SYNJ2BP increases the stability of SNAI1 via the phosphorylation of AKT.

## DISCUSSION

The present study demonstrated that the level of SYNJ2BP expression may be used as an indicator to distinguish between breast carcinomas and normal tissues, since it was found to correlate with breast carcinoma progression in breast tumor samples. IHC analysis showed that SYNJ2BP expression was significantly different when compared between normal and breast carcinoma tissues. Particularly strong SYNJ2BP expression was observed in high grade breast carcinomas (*p* < 0.0001) (Figure 1), suggesting that SYNJ2BP could promote the migration and invasion of breast cancer cells. It is now widely accepted that the classical EMT phenotype can strongly promote cancer progression, invasion and metastasis. The overexpression of SYNJ2BP in breast cancer cell lines induced cytoskeletal rearrangement in the whole cell, reducing cell-cell contact and by promoting cell migration and EMT (Figures 2 and 3), suggesting that SYNJ2BP changed the cell EMT phenotype to promote breast cancer cell motility, migration and invasion.

Activation of the EMT-inducing transcription factor, SNAI1, can be clearly demonstrated in invasive carcinomas [27]. In previous studies, we found that SNAI1 cooperates with ERα and DACH1 to regulate E-cadherin expression and EMT in breast cancer cells by binding with the E-box of the E-cadherin promoter [28, 29]. In this study, SYNJ2BP was shown to enhance breast tumor metastasis and EMT by activating SNAI1 (Figure 4). EMT progression is regulated by the PI3K/AKT, ERK, p38 and JNK signaling pathways to induce SNAI1 activated [13]. The export of SNAI1 is controlled by GSK3β, and stabilization of SANI1, a repressor of E-cadherin in the nucleus, which leads to the promotion of EMT. By blocking different pathways with small molecule inhibitors, we showed that SYNJ2BP regulated the EMT process by activating the PI3K/AKT/GSK3β/SNAI1 pathways. PI3K is responsible for coordinating a diverse range of cellular functions, including proliferation, cell survival, degradation, vesicular trafficking and cell migration [9]. In our studies, SYNJ2BP inhibited PTEN function and activated signaling pathways downstream of PI3K. Activated AKT, which is phosphorylated by PI3K, was also been shown to phosphorylate GSK3β directly (Figure 4). Phosphorylation of GSK3β is deregulated by the ubiquitin-proteasome pathway [11]. Therefore, SNAI1 was not moved out of nucleus to the cytoplasm by GSK3β, and nuclear SNAI1 enhanced the progression of EMT (Figure 4). SYNJ2BP was able to directly regulate GSK3β phosphorylation by influencing the PI3K/AKT pathway to enhance breast cancer cell metastasis (Figure 4).

In addition, SYNJ2BP has also been shown to regulate angiogenesis and promote Notch signaling through the stable Notch ligands, DLL1 and DLL4, in endothelial cells [30, 31]. A role for Notch is well established in hematological malignancies, but in solid tumors seems to be highly dependent on the individual context. Previous research has suggested that Notch acts as an oncogene in some cancers, but as a tumor suppressor in others [32]. Therefore, SYNJ2BP plays different functions in breast cancer and hepatocellular carcinoma. Notch signaling is also known to disrupt GSK3β/SNAI1 interactions, and increases SNAI1 stability to initiate EMT [13, 33]. Furthermore, SYNJ2BP reduces Activin signaling by interacting with ActRII (Activin type II receptor). Activin is a member of the TGFβ superfamily and is also one of the important factors underlying the induction of EMT in cancer cells [34]. Reduction of Activin signaling mediated by SYNJ2BP suggested that SYNJ2BP may affect EMT through Notch, PI3K or the Activin signaling pathway in different types of tumor.

The localization of SYNJ2 to the leading edge of cells depends on dynamin, with SYNJ2BP acting as a SYNJ2 binding protein. The oncogenic activity of SYNJ2 relates to its ability to dephosphorylate phosphoinositides and reduce polarization signals. PI3K produces PI(3,4,5)P3, and SYNJ2 dephosphorylates PI(3,4,5)P3 to produce PI(3,4)P2, while PTEN dephosphorylates PI(3,4,5)P3 to produce PI(4,5)P2. Thus, PTEN also inhibits SYNJ2 activity, and SYNJ2BP promotes the degradation of PTEN to promote the progression of tumors induced by both PI3K and SYNJ2. In a previous study, Li found that SYNJ2BP was expressed more frequently in mucinous adenocarcinoma [17]. This suggests that SYNJ2BP may play an important role in cancer progression. In the present study, we found that SYNJ2BP regulated the EMT phenotype to promote breast cancer cell metastasis, and thus identified a key mechanism of tumor metastasis.

The deletion and mutation of PTEN is a key step in the development of many cancers, including breast cancer [35]. In previous studies, we found that PTEN was a main player in the regulation of PI3K signaling [36], and the stability of PTEN was regulated by ubiquitination through NEDD4 and USP13 [37, 38]. In this present study, we found that PTEN was degraded by lysosome-associated pathways, besides the ubiquitination proteasome pathway. We observed a high level of overlap between the staining of PTEN and that of lysosomes in the cytoplasm when SYNJ2BP was overexpressed in cells. The co-localization of PTEN with lysosomes was further enhanced in serum-starved cells (Figure 5), suggesting that changes in PTEN expression, induced by the overexpression of SYNJ2BP, may be attributed to lysosome degradation.

There are two major mechanisms for protein degradation: the autophagy-lysosome and the ubiquitin-proteasome pathway. As far as we know, the lysosome pathway is a non-specific degradation process. Large protein complexes, or long-lived and stable proteins, can be degraded by the lysosome proteolysis pathway. Only the chaperone-mediated autophagy-lysosome pathway can specifically degrade substrate proteins which are bound to the protein–molecular chaperone (heat-shock cognate protein of 70 kDa, hsc70) complex and lysosomal membrane protein LAMP-2, which must also be translocated across lysosomal membranes for their degradation [39]. The autophagy-lysosome is thought to mainly represent a non-selective degradation pathway for long lived proteins and some organelles. However, in this study we showed that SYNJ2BP may act as an adaptor that specifically binds with PTEN and then mediates the degradation of PTEN by the lysosome pathway. A thorough knowledge of the mechanisms involved in the targeting of substrates to the lysosome will provide new clues to novel mechanisms of protein degradation and tumor metastasis.

SYNJ2BP plays a pivotal role in vesicular trafficking and the recycling of membrane protein and cellular motility, particularly in breast cancer metastasis [10]. SYNJ2BP is localized to the plasma membrane, where it interacts with ActRII and RALBP1 (Ral binding protein 1) and changes the localization of ActRII through endocytosis [14]. SYNJ2BP is localized to the mitochondrial outer membrane and mediates the recruitment of synaptojanin 2A to the mitochondria, and maintains its distribution within the mitochondria [15]. Our current data showed that SYNJ2BP was localized to the autophagy-lysosomal membrane with LC3-II, which then mediated the recruitment of PTEN to the lysosome followed by its degradation. The autophagosome for PTEN degradation is a typical autophagosome combined with LC3-II and p62, and the formation of autophagic vesicles relies on ATG5. SYNJ2BP can be combined with specific proteins such as PTEN, to induce its degradation by autophagy-lysosome pathway.

In summary, SYNJ2BP plays a variety of cellular functions by combining with different proteins, including binding proteins for endocytosis localization, cycling and degradation. In addition, SYNJ2BP may also regulate the PI3K signal pathway through the autophagy-lysosome-associated degradation of PTEN, suggesting that SYNJ2BP might be closely associated with lysosome membrane proteins. This not only demonstrated a novel degradation mechanism for PTEN by the SYNJ2BP-mediated pathway, but also provided new insights into the mechanisms of cancer and highlighted a new potential target for clinical detection.

## MATERIALS AND METHODS

### Cell culture and transfection

Human breast cancer cell lines (MCF-7, T47D, ZR-75–30 & MDA-MB-231), and mouse mammary tumor cell line 4T1 were cultured as previously described [28, 40–42]. Cells were grown at 37°C in a humidified 5%–CO_2_ atmosphere and transfected using Lipofectamine 2000 (Invitrogen, Auckland, New Zealand) according to the manufacturer's specifications.

### Plasmids and reagents

Homo sapiens (human) SYNJ2BP (GENE ID 55333) gene were cloned into pcDNA and pEGFP vector by PCR using the primers 5′- GGAATTCTATGA ACGGAAGAGTG-3′ (forward), and 5′- GGGGTACCTC AAGTTGTCTTAGTC-3′ (reverse). SYNJ2BP shRNA expression vectors pRNAT-U6.1 vector (GenScript, Piscataway, NJ, USA), and shSYNJ2BP#1, shSYNJ2BP#2, and shSYNJ2BP#3 were used for SYNJ2BP knockdown study. The primers of shSYNJ2BP#1, #2, and #3 sequences were as follows:5′-GCCGCATCAAAGAAAATGGG-3′, 5′-GTAGACCTCTTTCGTAATGC-3′ and 5′- GAGAAT AAAATATCTTTAGA-3′. ShATG5 sequences were as follows: 5′-GGCATTATCCAATTGGTTTA-3′ [43]. ShCtrl and shSNAI1 were acquired as previously described [29].

Antibodies used were as follows: anti–glyceraldehyde-3-phosphate dehydrogenase (anti-GAPDH) (Sigma, Saint Louis, MO, USA); anti-Snail (Thermo fisher, Massachusetts, USA), anti-GSK3β, anti-GSK3β (phospho-Ser9), anti-Akt, anti-Akt (phospho-Ser473), anti-N-cadherin, anti-PTEN (Santa Cruz, Dallas, CA, USA), and anti–E-cadherin; anti-Cytokeratin 18; anti-vimentin (Abcam, Cambridge, MA, USA) [28]; anti–SYNJ2BP, anti-p62 and anti-ATG5 (Proteintech, SanYing Biotechnology, China); anti-LC3-II and Chloroquine diphosphate salt (Sangon, Sangon Biotech, China). Rabbit polyclonal anti-GFP and mouse monoclonal anti-Flag (M2) antibodies, cycloheximide, LY294002 and LiCl were purchased from Sigma Aldrich (Saint Louis, MO, USA). MG132 was obtained from Merck (Merck KGaA, Gemany).

### Luciferase reporter assay, immunofluorescence and MTT

Luciferase reporter assay, Immunofluorescence and MTT assays were performed as described in our previous study [28]. E-cadherin promoter sequences were cloned into the pGL3 vector as previous described [29]. The slides were incubated with antibody directed against LC3-II as previous described [28]. MTT assay was performed according to the manufacturer's protocol (KeyGen, Nanjing, China) as previous described [44].

### GST pull-down assays

GST alone and GST-SYNJ2BP fusion proteins were expressed in *Escherichia coli* BL21 cells and purified by means of the Pierce GST Spin Purification Kit (Thermo-Pierce, Rockford, IL, USA) as described in our previous study [28].

### Trans-well and wound-healing

For trans-well migration assay, cells (1 × 10^5^) were placed in the upper chamber of a trans-well with serum free medium. For trans-well invasion assay, the trans-well membranes were pre-coated with Matrigel (BD Transduction, Franklin Lake, NJ). After 12 h, cells were fixed for 20 min in 4% glutaraldehyde prepared in PBS. Staining was carried out for 30 min in 0.1% crystal violet and 2% ethanol. The number of migratory cells was counted under an inverted microscope (Nikon TE2000-U). Wound-healing assay was done as described previously [29, 45].

### Western blot, immunoprecipitation and immunohistochemistry

Western blot, immunoprecipitation (IP) and immunohistochemistry (IHC) were performed as previously described [36, 46, 47]. Thirty-nine breast carcinoma tissue samples and five normal/pericarcinomatous tissue samples used for immunohistochemical analysis were obtained from Qiqihar Medical University. All individuals who donated the tissues for this study gave their consent in written form. All treatments and experimental protocols for this study were performed in accordance with established guidelines and regulation by the Biological and Medical Ethics Committee of Dalian University of Technology. The Biological and Medical Ethics Committee of Dalian University of Technology gave approval for the animal experiments.

### Lung metastasis assay *in vivo*

All animal treatments and experimental protocols for this study were performed in accordance with established guidelines and regulation by the Biological and Medical Ethics Committee of Dalian University of Technology. BALB/c mice (6–8 weeks) were purchased from the Laboratory Animal Center of Dalian Medical University. 4T1 cells were stably transfected with pcDNA-SYNJ2BP or pcDNA, and 100 μl of these cells (10^6^ cells) suspended in normal saline was injected into the tail vein of BALB/c mice. Control BALB/c mice were injected with 100 μl normal saline only. Each group consisted of five animals. On the 10th day, the animals were killed under anesthesia and the sizes and weights of the lungs were recorded and subjected to further analysis (paraffin embedding and digestion for subsequent extraction of protein) as previously described [28, 44].

### Statistical analysis

All statistical analyses of the data were performed with ANOVA. Data were expressed as means ± S.D., and significance was considered at either the *P* < 0.05 or *P* < 0.01 level. A Fisher's exact test was used to examine the correlation between SYNJ2BP, p-AKT and SNAI1 expression in breast cancer tissues from the 39 patients.
